# Nonproteolytic K29-Linked Ubiquitination of the PB2 Replication Protein of Influenza A Viruses by Proviral Cullin 4-Based E3 Ligases

**DOI:** 10.1128/mBio.00305-20

**Published:** 2020-04-07

**Authors:** Marwah Karim, Elise Biquand, Marion Declercq, Yves Jacob, Sylvie van der Werf, Caroline Demeret

**Affiliations:** a Unité de Génétique Moléculaire des Virus à ARN, Département de Virologie, Institut Pasteur, Paris, France; b UMR 3569, Centre National de la Recherche Scientifique (CNRS), Paris, France; c Université de Paris, Paris, France; Icahn School of Medicine at Mount Sinai

**Keywords:** K29-linked ubiquitination, PB2 replication protein, cullin-based E3 ligases, influenza virus, nonproteolytic ubiquitination, post-translational modification, ubiquitination

## Abstract

The multifunctional nature of viral proteins is essentially driven by posttranslational modifications (PTMs) and is key for the successful outcome of infection. For influenza A viruses (IAVs), a composite pattern of PTMs regulates the activity of viral proteins. However, almost none are known that target the PB2 replication protein, except for inducing its degradation. We show here that PB2 undergoes a nonproteolytic ubiquitination during infection. We identified E3 ubiquitin ligases catalyzing this ubiquitination as two multicomponent RING-E3 ligases based on cullin 4 (CRL4s), which are both contributing to the levels of ubiquitinated forms of PB2 in infected cells. The CRL4 E3 ligase activity is required for the normal progression of the viral cycle and for maximal virion production, indicating that the CRL4s mediate a ubiquitin signaling that promotes infection. The CRL4s are recruiting PB2 through an unconventional bimodal interaction with both the DDB1 adaptor and DCAF substrate receptors. While able to bind to PB2 when engaged in the viral polymerase complex, the CRL4 factors do not alter transcription and replication of the viral segments during infection. CRL4 ligases catalyze different patterns of lysine ubiquitination on PB2. Recombinant viruses mutated in the targeted lysines showed attenuated viral production, suggesting that CRL4-mediated ubiquitination of PB2 contributes to IAV infection. We identified K29-linked ubiquitin chains as main components of the nonproteolytic PB2 ubiquitination mediated by the CRL4s, providing the first example of the role of this atypical ubiquitin linkage in the regulation of a viral infection.

## Abstract (precis)

Successful infection by influenza A virus, a pathogen of major public health importance, involves fine regulation of the multiple functions of the viral proteins, which often relies on post-translational modifications (PTMs). The PB2 protein of influenza A viruses is essential for viral replication and a key determinant of host range. While PTMs of PB2 inducing its degradation have been identified, here we show that PB2 undergoes a regulating PTM signaling detected during infection, based on an atypical K29-linked ubiquitination and mediated by two multicomponent E3 ubiquitin ligases. Recombinant viruses impaired for CRL4-mediated ubiquitination are attenuated, indicating that ubiquitination of PB2 is necessary for an optimal influenza A virus infection. The CRL4 E3 ligases are required for normal viral cycle progression and for maximal virion production. Consequently, they represent potential candidate host factors for antiviral targets.

## INTRODUCTION

Ubiquitination of proteins is a versatile post-translational modification involved in virtually all cell processes (1). A primary function of protein ubiquitination is to target ubiquitinated proteins to the proteasome where they are degraded, which is the predominant mechanism of protein turnover in cells. A number of nonproteolytic functions of ubiquitination exist, which basically regulate protein activity (2). The versatile functions of protein ubiquitination depend on the type of ubiquitin (Ub) linkages to the targeted protein, which is referred as the “ubiquitin code.” Ub contains seven lysine (K) residues that can be employed by ubiquitin ligases to generate different types of Ub linkages on the targeted proteins, which, in turn, will interact with different downstream cellular factors. It is well established that K48-based linkages target proteins to the proteasome for their degradation, while K63-based ubiquitin chains are the prominent nonproteolytic ubiquitin signals, primarily controlling protein trafficking or activity (3). The roles of other types of polyubiquitination, i.e., K6-, K27-, K29-, and K33-linked ubiquitin chains (also referred to as atypical polyubiquitin), remain more elusive. Nevertheless, there is growing evidence suggesting their importance in cell regulation (4, 5). Furthermore, an increasing complexity emerges in ubiquitin chain topologies, which turn out to frequently combine mixed lysine linkages in a single chain (5).

Protein ubiquitination involves the serial action of an E1-activating enzyme that activates the ubiquitin molecule, followed by the transfer of ubiquitin to an E2 ubiquitin-conjugating enzyme and subsequently ubiquitin transfer to a substrate protein by an E3 ligase (6). The E3 ubiquitin ligases determine substrate specificity for ubiquitination. They are categorized, based on the mechanism of ubiquitin transfer to substrate proteins, into RING, HECT (homologous to the E6AP carboxyl terminus), and RBR (RING-between-RING) families (7). The RING E3 ligases are working either as monomers or as multiprotein complexes for the cullin-based RING-E3 ligases (CRLs) (7). In CRL ubiquitin ligases based on cullin 4 (CRL4s), DDB1 acts as an adaptor between the cullin 4 and substrate recognition factors (SRFs), which are typical DWD-containing proteins designated DCAF (DDB1-cullin 4-associated factors). The SRFs are responsible for substrate protein recruitment to the CRL4 complexes for ubiquitination (8).

Post-translational modifications (PTMs) of proteins enhance the multifunctional nature of viral proteins, thus playing an important role in the orchestration of viral processes and in cellular responses to infection, and are therefore essential to determine the successful outcome of an infection. For influenza A viruses (IAVs), diverse types of PTMs have been shown to tailor the functions of viral proteins during the replicative cycle, including phosphorylation, ISGylation, glycosylation, palmitoylation, acetylation, SUMOylation, neddylation, ADP-ribosylation, and ubiquitination (reviewed in reference 9). Indeed, ubiquitination of several viral proteins has been reported to support different phases of the influenza virus life cycle. For instance, ubiquitination of the matrix protein M1 by the HECT E3 ubiquitin ligase Itch assists the release of viral particles from the endosome during virus entry (10), and ubiquitination of the M2 protein is required for the production of infectious virions (11). The CNOT4 E3 ligase mediates ubiquitination of the nucleoprotein NP and supports virus replication (12). On the other hand, the RING-E3 ligases TRIM22 and TRIM32 have been shown to ubiquitinate NP and PB1 proteins respectively, and target them for proteasomal degradation, thereby restricting IAV infection (13, 14). A composite pattern of viral protein ubiquitination seems therefore to be at play during influenza A virus infection.

Surprisingly, only a few, if any, PTMs are described so far that regulate the function of the PB2 replication factor, which is an essential component of the viral transcription/replication machinery and a key determinant of host range. Neddylation and ADP-ribosylation have been reported on PB2, both of which are inducing its degradation, resulting in inhibition of viral replication (15, 16). In addition, one study has reported the ubiquitination of all the replication proteins of IAV, including PB2, independently of their degradation, and the stimulation of the viral polymerase function by ubiquitin overexpression, the mechanism of which remains elusive (17).

We show here that the PB2 protein is ubiquitinated during infection and this ubiquitination signal is mainly composed of atypical K29 linkages, which does not lead to its degradation. We identified two multicomponent RING-E3 ligases based on cullin 4, CRL4^D12L1^ and CRL4^D11^ containing the DDB1 adaptor and either DCAF12L1 (D12L1) or DCAF11 (D11) as substrate recognition factors, respectively, that catalyze PB2 ubiquitination in an infectious context. The activity of these E3 ligases is required for the normal progression of the viral cycle as well as for maximal virion production, indicating that they are mediating a ubiquitin signaling required for optimal IAV infection. Recombinant viruses impaired for CRL4-mediated ubiquitination are attenuated, suggesting that the ubiquitination on PB2 contributes to the productive viral cycle. This is the first example of the modification of a viral protein by K29-linked ubiquitin chains, illustrating a role of this atypical linkage in pathogen regulation.

## RESULTS

### Knockdown of CRL4 factors hinders IAV life cycle.

We have recently determined the interplay that is established between the PB2 protein of influenza A viruses and the human ubiquitin proteasome system (UPS) through pairwise interactions (18). Three factors belonging to the cullin 4-based RING E3 ligase (CRL4) family, i.e., the DDB1 adaptor and two SRFs, DCAF12L1 and DCAF11, were identified as high-confidence PB2 interactors. Binary interaction of each of these factors (designated CRL4 factors) with the PB2 protein of several IAV strains has been validated in a split-luciferase assay (18). In addition, coimmunoprecipitation (co-IP) experiments with ectopically expressed Strep-tagged CRL4 factors and 3×Flag-H1N1_WSN_ PB2 confirm their direct interaction with PB2 (see Fig. S1A in the supplemental material). Preliminary functional studies made with small interfering RNA (siRNA) pools have indicated that the depletion of CRL4 factors affects virus production 24 h postinfection (18). Using deconvoluted siRNA from the pools used in our initial study, we found that all single siRNAs for DDB1 efficiently knocked down DDB1 expression and reduced viral titers, while for DCAF12L1 one siRNA (siRNA 1) sufficiently silenced gene expression to affect viral production, and for DCAF11, two siRNAs (siRNAs 1 and 3) were effective in gene silencing and reducing viral production (Fig. S1B). The effects of CRL4 factors knocked down on viral production ranged from 5- to 10-times reduction, in accordance with our previous results (18). For each CRL4 factor, one single siRNA with no major effect on cell viability (not shown), siDDB1-2, siDCAF12L1-1, and siDCAF11-1, was selected for the subsequent studies.

10.1128/mBio.00305-20.1 FIG S1 (A) Interaction of PB2 with the CRL4 factors. HEK293T cells were transfected with Strep-DDB1, Strep-DCAF12L1 (D12L1), Strep-DCAF11 (D11), or Strep-empty, together with 3×Flag-PB2 or 3×Flag-empty. At 24 h posttransfection, cells were lysed and Strep-Tactin pulldown was performed. Proteins in the pulled fraction and in 1/10 of whole-cell extract (inputs) were analyzed by Western blotting as shown. (B) Knockdown efficiency and effect on viral titers of deconvoluted siRNA directed against DDB1, DCAF12L1, and DCAF11. A549 cells were transfected with the indicated siRNA. (Left) At 48 h after siRNA transfection, the knockdown efficiency was monitored by RT-qPCR for DCAF12L1 and DCAF11, on total RNA extracted with the RNeasy minikit (Qiagen) and using the LightCycler RNA amplification kit SYBR green I (Roche). For DDB1, knockdown efficiency was monitored by measuring luciferase activity after cotransfection of siDDB1 with expression plasmids for DDB1-*Gaussia* luciferase fusion protein or for the unfused *Gaussia* luciferase, as described in reference 18. (Right) At 48 h after siRNA transfection, cells were infected with H1N1_WSN_ at an MOI of 0.0001 for 24 h. Viral titers were determined by plaque-forming assay. Data represent means ± SD for three independent experiments, and the significance was measured using one-way ANOVA. Download FIG S1, PDF file, 0.4 MB. Copyright © 2020 Karim et al. 2020 Karim et al. This content is distributed under the terms of the Creative Commons Attribution 4.0 International license.

**FIG 1:**
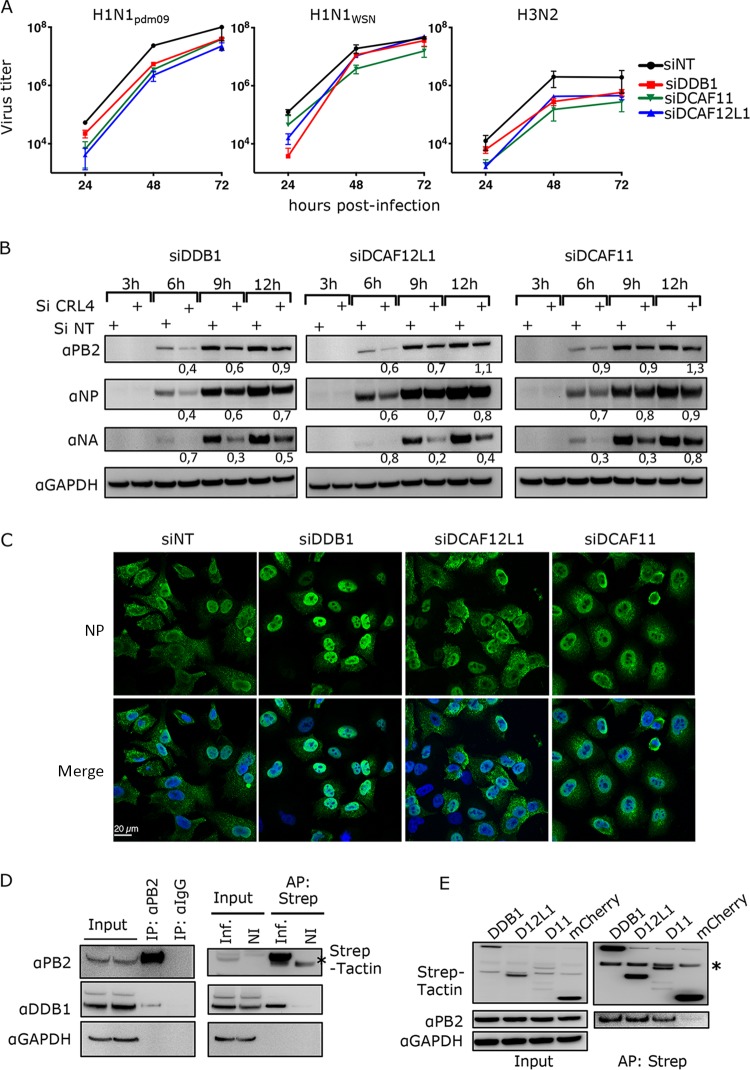
Involvement of CRL4 factors in IAV infection. (A) A549 cells were transfected with siRNA nontarget (NT) or siRNA targeting CRL4 factors (si target) for 48 h and then infected at an MOI of 0.0001 PFU/cell (H1N1_WSN_) or 0.001 PFU/cell (H1N1_pdm09_ and H3N2). Viral titers were determined by plaque-forming assay at the indicated time points. Statistical significances are given in Table S1. (B) A549 cells transfected with the indicated siRNA for 48 h were infected with H1N1_pdm09_ at an MOI of 3 PFU/cell. Total cell lysates were prepared at the indicated time postinfection and analyzed by Western blotting using the indicated antibodies. The relative amounts of viral proteins in siRNA-treated samples compared to the corresponding siRNA nontarget (NT) samples are indicated. (C) A549 cells transfected with the indicated siRNA for 48 h were infected with H1N1_WSN_ at an MOI of 5 PFU/cell. At 6 h postinfection, cells were fixed, permeabilized, and stained with an anti-NP antibody (green) and with Hoechst 33342 (blue). Representative images of NP localization are shown. Quantification of the NP labeling is provided in Fig. S2C. (D) HEK293T cells were noninfected (NI) or infected with H1N1_WSN_ (left) or H1N1_WSN_-PB2-Strep (right) virus at an MOI of 3 for 6 h. Cell lysate was subjected to anti-PB2 antibody and IgG (left) or to Strep-Tactin pulldown (right). Proteins in the pulled fractions and in 1/10 of whole-cell extract (inputs) were assessed in an immunoblot assay as indicated. (E) HEK293 cells stably expressing Strep-DDB1, Strep-DCAFs (D12L1 for DCAF12L1 or D11 for DCAF11), or Strep-mCherry (control) were infected with H1N1_WSN_ at an MOI of 3 for 6 h and subjected to Strep-Tactin pulldown. The PB2 protein copulled with the Strep-CRL4 factors was detected using anti-PB2 antibody. One-fourth of whole-cell extract was used to detect the Strep-CRL4 factors in the input since they were undetected in a 1/10 fraction, indicating a low level of Strep fusion expression. The asterisk indicates an aspecific band.

To further analyze the role of these factors in IAV infection, we measured the kinetics of virus production upon siRNA knockdown of each DDB1, DCAF11, and DCAF12L1 factor using the fully lab-adapted strain H1N1_WSN_, and seasonal H1N1_pdm09_ and H3N2 strains, which were recently adapted to *in vitro* infection (18). Silencing of CRL4 factors moderately but significantly dampened IAV production compared to the control nontarget (NT) (Fig. 1A and Table S1). While H1N1_WSN_ recovered at normal or nearly normal levels at the later infection times, a reduction in virus production was still observed for the seasonal strains.

10.1128/mBio.00305-20.10 TABLE S1 Statistics of the viral titers upon siRNA knockdown of the CRL4 factors. *P* values were calculated using ordinary one-way ANOVA with Dunnett’s multiple-comparison test. Download Table S1, PDF file, 0.04 MB. Copyright © 2020 Karim et al. 2020 Karim et al. This content is distributed under the terms of the Creative Commons Attribution 4.0 International license.

10.1128/mBio.00305-20.2 FIG S2 Effect of CRL4 factors on IAV infection. (A) Effect of the knockdown of CRL4 factors on the accumulation of viral proteins in H1N1_WSN_-infected cells. A549 cells were transfected for 48 h with control nontarget (NT) or siRNA for DDB1, DCAF12L1, and DCAF11 as indicated and subsequently infected with H1N1_WSN_ at an MOI of 3 PFU/cell. Total cell lysates were prepared at the indicated time postinfection and analyzed by Western blotting using antibodies as shown. (B) A549 cells transfected with control NT or siRNA specific for DDB1, DCAF12L1, and DCAF11 for 48 h were infected with H1N1_WSN_ at an MOI of 5 PFU/cell. At 6 h postinfection, cells were fixed, permeabilized, and stained with anti-PB2 antibody (red) and with Hoechst 33342 (blue). (C) Quantification of the NP and PB2 labeling in the nucleus and cytoplasm from immunofluorescence images (Fig. 1C and panel B of this figure, respectively). The percentage of nuclear and cytoplasmic labeling is given for each condition. Download FIG S2, PDF file, 0.4 MB. Copyright © 2020 Karim et al. 2020 Karim et al. This content is distributed under the terms of the Creative Commons Attribution 4.0 International license.

To explore the effect of CRL4 factors on a single viral cycle, we examined the accumulation of the viral proteins in CRL4 factor-depleted A549 cells infected at a high multiplicity of infection (MOI) with H1N1_pdm09_ or H1N1_WSN_ at different time points. For both strains, an overall slowdown in viral protein accumulation was detected (Fig. 1B and Fig. S2A), reminiscent of a delayed viral cycle. The viral cycle progression was next assessed by staining viral NP and PB2 proteins at 6 h postinfection in siRNA-treated A549 cells. In control siRNA-treated cells, NP staining either was cytoplasmic or formed a ring in the nucleus, reflecting the stage of nuclear export of the vRNPs to the cytoplasm (Fig. 1C). In cells treated with siDDB1, NP staining was nuclear and homogenous, reflecting that vRNP export did not begin (Fig. 1C). In the DCAF-depleted cells, the nuclear NP staining was also enhanced relative to the cytoplasmic staining, while to a lesser extent than in DDB1-depleted cells. Quantification of NP localization indicated that depletion of each CRL4 factor increased the proportion of nuclear staining compared to NT siRNA (Fig. S2C). Indeed, PB2 localized both in the nucleus and in the cytoplasm in siNT-treated cells, whereas it accumulated primarily in the nuclei upon silencing of the CRL4 factors (Fig. S2B and C). Altogether, these results indicate that the depletion of the CRL4 factors interferes with the normal progression of the viral cycle, causing a delay that is detected at the levels of both viral protein accumulation and subcellular distribution of vRNPs, and leads to a reduced production of IAV infectious virions.

### CRL4 factors interact with PB2 during influenza A virus infection.

We next addressed the interaction of PB2 with endogenous DDB1 during infection. HEK293T cells were either mock infected or infected with H1N1_WSN_ or with H1N1_WSN_ expressing a Strep-tagged PB2 protein (H1N1_WSN_-PB2-Strep) at an MOI of 3. Pulldown of PB2 was carried out using anti-PB2 antibodies (H1N1_WSN_) or Strep-Tactin Sepharose beads (H1N1_WSN_-PB2-Strep), and then cells were probed for DDB1 by immunoblotting. The DDB1 copurified with untagged PB2 and with PB2-Strep, while it was absent in controls (Fig. 1D), indicating that the interaction between PB2 and DDB1 can be detected during infection. To establish whether the interaction of PB2 with the DCAFs can also be detected in an infectious context, we constructed HEK293 cells stably expressing Strep-tagged DCAF12L1 or DCAF11 owing to the lack of available antibodies detecting the endogenous proteins. Cells stably expressing Strep-DDB1 and Strep-mCherry were also produced. In cells stably expressing the Strep-CRL4 factors infected by H1N1_WSN_, the PB2 protein copurified with both DCAF12L1 and DCAF11, as well as with DDB1 as expected from the above-described experiments. In contrast, almost no PB2 protein was pulled with mCherry (Fig. 1E). The association of the three CRL4 factors with PB2 is therefore detected during infection, most likely as a result of a direct interaction between PB2 and the CRL4 factors (18) (Fig. S1A).

### PB2 associates with CRL4 E3 ligase complexes containing DDB1-DCAF12L1 or DDB1-DCAF11.

**FIG 2:**
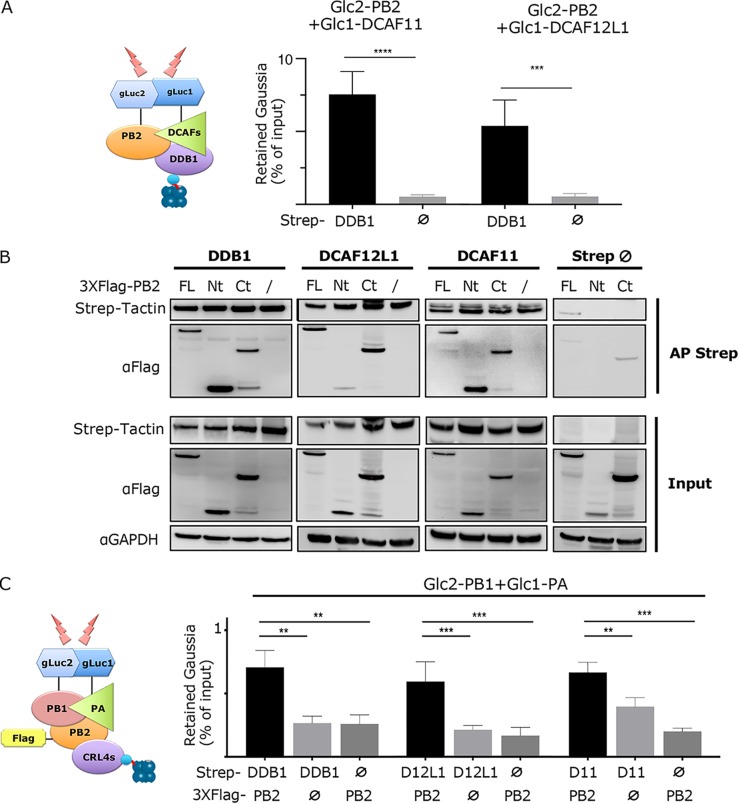
Interaction between the CRL4 complexes and the viral polymerase. (A) Detection of DDB1-PB2-DCAF triple complexes. A schematic of the triple-complex pulldown is given where Strep tag is represented by a cyan sphere and the Strep-Tactin beads are represented by blue spheres. The Gluc2-PB2, Gluc1-DCAFs (either DCAF12L1 or DCAF11), and Strep-DDB1 or Strep-empty expression plasmids were cotransfected in HEK293T cells. At 24 h posttransfection, cell lysates were subjected to pulldown using Strep-Tactin Sepharose beads. The *Gaussia* luciferase activity retained on the beads was expressed as the proportion of *Gaussia* activity in the whole-cell lysate (% of input). The *Gaussia* activity in the whole-cell lysate, representing the level of interacting protein pair, is given in Fig. S3A. Data represent means ± SD for three experiments, and significance was measured using a two-tailed unpaired *t* test (nonsignificant [ns], *P* > 0.05; *, *P* ≤ 0.05; **, *P* ≤ 0.01; ***, *P* ≤ 0.001; ****, *P* ≤ 0.0001). (B) Interaction domains of PB2 with the CRL4 factors. HEK293T cells were transfected with Strep-DDB1, Strep-DCAF12L1, or Strep-DCAF11 together with 3×Flag-PB2 full-length (FL), N-terminal (Nt), or C-terminal (Ct) domains or with 3×Flag-empty expression plasmids. At 24 h posttransfection, cells were lysed and Strep-Tactin pulldown was performed. Proteins in the pulled fraction and in 1/10 of whole-cell extract (inputs) were analyzed by Western blotting as shown. (C) Interaction of CRL4 factors with the RNA-dependent RNA polymerase (RdRP). A schematic of the quaternary-complex pulldown is given. HEK293T cells were cotransfected with Strep-DDB1, Strep-DCAF12L1, Strep-DCAF11, or Strep-empty vector along with either 3×Flag-PB2 or 3×Flag-empty and with Gluc1-PA and PB1-Gluc2. At 24 h posttransfection, cells were lysed and subjected to pulldown using Strep-Tactin Sepharose beads. The retention of *Gaussia* activity on the beads was expressed as the proportion of the *Gaussia* activity in the whole-cell lysate (% of input). The *Gaussia* activity in the whole-cell lysate before pulldown, representing the level of interacting PB1/PA dimer, is given in Fig. S3C. Data represent means ± SD for three independent experiments, and the significance was measured using one-way ANOVA.

We next explored whether PB2 binds to the DDB1, DCAF12L1, and DCAF11 as part of E3 ligase CRL4 complexes, i.e., if each of the DCAFs can form a triple complex with DDB1 and PB2 as expected for an SRF. In order to test this, we used a Gaussia princeps luciferase complementation assay (GPCA) to detect the reconstitution of luciferase activity when proteins expressed in fusion with the Gluc1 and Gluc2 complementary fragments of the enzyme are brought into close proximity (18, 19). To assess triple complex formation, the DCAFs (either DCAF12L1 or DCAF11) and PB2, fused to Gluc1 and Gluc2 fragments, respectively, were coexpressed with the Strep-tagged DDB1 or Strep alone. A Strep pulldown was performed, and the copurified reconstituted *Gaussia* luciferase activity was measured and related to the *Gaussia* activity in the whole-cell extract prior to pulldown. Significantly more PB2-DCAF12L1- and PB2-DCAF11-mediated luciferase activities were retained on the beads in the presence of DDB1 compared to the Strep-empty control (Fig. 2A and Fig. S3A). These triple-complex analyses strongly suggest that PB2 can associate with DDB1-DCAF complexes, which compose E3 ligase complexes. To confirm the protein-protein interactions involved, HEK293T cells were transfected with expression constructs for Strep-DDB1, in combination with either DCAF12L1 or DCAF11 fused to Gluc1 and with 3×Flag-PB2 or 3×Flag-empty. Strep pulldown was performed, and proteins coprecipitated with Strep-DDB1 were analyzed. As expected from their role as SRFs, interaction between DDB1 and DCAF12L1 or DCAF11 was detected in the absence of PB2 (Fig. S3B). The PB2 protein and each DCAF were copulled with Strep-DDB1 (Fig. S3B), strengthening our previous results.

10.1128/mBio.00305-20.3 FIG S3 (A) *Gaussia* activity in the whole-cell lysates before pulldown of the triple complexes (related to Fig. 2A), reflecting the level of interacting protein pairs. (B) Pulldown analysis of CRL4 complex-PB2 interactions. HEK293T cells were transfected with Gluc1-DCAFs (either DCAF12L1 or DCAF11) together with Strep-DDB1 or Strep-empty and 3×Flag-PB2 or 3×Flag-empty expression plasmids. At 24 h posttransfection, cells were lysed and Strep-Tactin pulldown was performed. Proteins in the pulled fraction and in 1/10 of whole-cell extract (inputs) were analyzed by Western blotting as shown. (C) *Gaussia* activity in the whole-cell lysates before pulldown of the quaternary complexes (related to Fig. 2C), reflecting the level of interacting PB1/PA. Download FIG S3, PDF file, 1.2 MB. Copyright © 2020 Karim et al. 2020 Karim et al. This content is distributed under the terms of the Creative Commons Attribution 4.0 International license.

The PB2 protein can exist in different forms during the IAV life cycle, i.e., a free form or complexed with PB1 and PA in the heterotrimeric viral polymerase complex. The structure of the polymerase complex shows that the N-terminal one-third of PB2 forms an invariant catalytic core together with PB1 and the C-terminal domain of PA, while the C-terminal two-thirds of PB2 is flexibly linked to the polymerase core (20). We studied which of these PB2 regions is interacting with the CRL4 factors by pulldown experiments. The 247 N-terminal (PB2-Nt) and the 500 C-terminal (amino acids [aa] 248 to 760, PB2-Ct) residues were fused to 3×Flag and coexpressed with Strep-tagged CRL4 factors. Both PB2-Nt and PB2-Ct domains were efficiently pulled with Strep-DDB1 and with Strep-DCAF11, while for Strep-DCAF12L1 only PB2-Ct was significantly pulled (Fig. 2B). These data indicate that the PB2 protein associates with DDB1 and DCAF11 through interfaces involving both its N- and C-terminal regions, while interaction with DCAF12L1 is mediated mainly by its C-terminal region.

### The CRL4 E3 factors can interact with the trimeric viral polymerase through PB2.

The polymerase-associated form is the main functional form of PB2 during IAV infection, mediating transcription and replication of the viral segments. To study the binding to CRL4 factors when PB2 is a part of the trimeric polymerase complex, GPCA-based quaternary protein complex experiments were conducted, in which the PB1-PA interaction was revealed by the *Gaussia* luciferase activity. Association of the interacting PB1-PA pair with Strep-tagged CRL4 factors was assessed by Strep pulldown, in the presence of coexpressed 3×Flag-PB2. Higher levels of PB1-PA-mediated luciferase activity were retained on the Strep-DDB1-, Strep-DCAF12L1-, or Strep-DCAF11-loaded beads when PB2 was coexpressed, compared to the controls without PB2. In the presence of PB2, Strep-empty led to retention of background levels of PB1/PA (Fig. 2C). These results suggest first that the CRL4 factors can associate with PB2 in the context of the trimeric polymerase complex and second that the recruitment of the CRL4 complex to this complex is mediated mainly by PB2.

### CRL4-based E3 ligases mediate PB2 ubiquitination.

**FIG 3:**
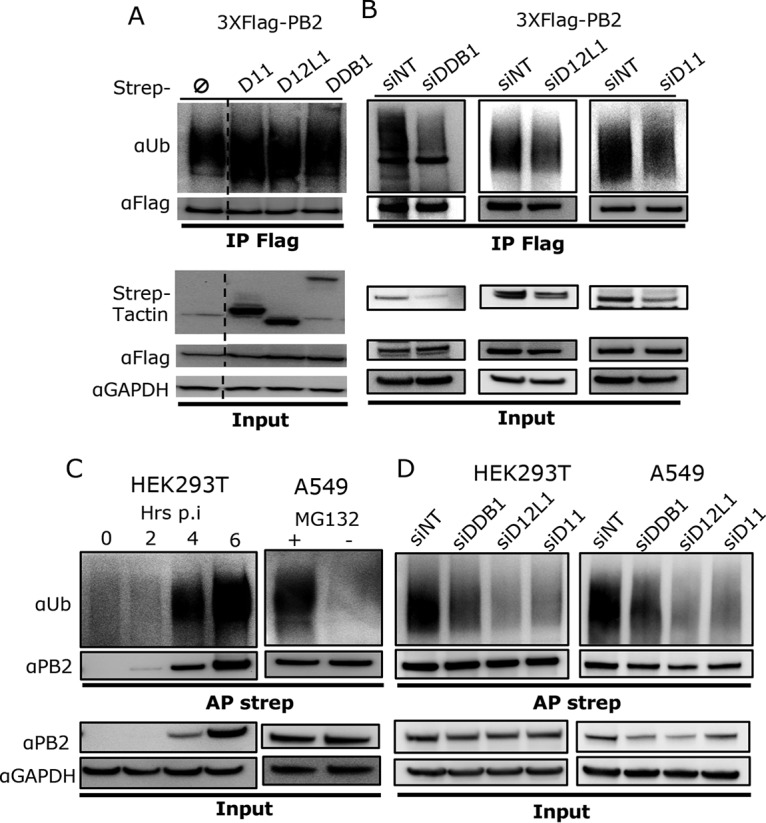
CRL4 E3 ligase complexes mediate PB2 ubiquitination. (A) HEK293T cells were transiently transfected with the indicated expression plasmids. At 36 h posttransfection, cells were treated with MG132 (10 μM) for 4 h. Anti-Flag immunoprecipitation (IP) was performed from cell lysates prepared under strong denaturing conditions (2% SDS), and the ubiquitinated forms of PB2 were detected using antiubiquitin antibody, followed by an anti-Flag immunoblot assay to detect PB2. Expression of Flag-PB2 and the Strep fusion proteins was monitored in cell lysate (Input). A dashed line marks that a lane from the initial membrane has been removed (not shown here). (B) HEK293 cells stably expressing Strep-DDB1, Strep-DCAF12L1 (D12L1), or Strep-DCAF11 (D11) were transfected with the indicated siRNA and 24 h later transfected with 3×Flag-PB2 for 36 h and treated with MG132 (10 μM) for 4 h. Cell lysate was processed for anti-Flag immunoprecipitation and immunoblotting as shown in panel A. (C) HEK293T cells were infected with H1N1_WSN_-Strep at an MOI of 3 for different times and treated with MG132 (10 μM) for 4 h before cell lysis (left). Similarly, A549 cells were infected with H1N1_WSN_-Strep at an MOI of 3 for 8 h and treated or not with 10 μM MG132 4 h before cell lysis (right). Strep-PB2 protein was pulled using Strep-Tactin Sepharose beads, and antiubiquitin and anti-PB2 immunoblotting assays were performed. (D) HEK293T and A549 cells transfected with the indicated siRNA for 48 h were infected with H1N1_WSN_-Strep at an MOI of 3 for 6 and 8 h, respectively, and treated with MG132 (10 μM) for 4 h before cell lysis. PB2-Strep pulldown and immunoblot assays were performed as described for panel C.

Having shown that the PB2 protein associates with DDB1-DCAF12L1 and DDB1-DCAF11 cocomplexes, we assessed whether these factors can ubiquitinate PB2. The level of PB2 ubiquitination in the presence or absence of CRL4 factors was first examined in a noninfectious context in HEK293T cells cotransfected with an H1N1_WSN_ PB2 Flag-tagged expression construct together with plasmids expressing Strep-tagged DDB1 or DCAFs or Strep-empty. Cells were treated for 4 h with MG132 proteasome inhibitor to detect endogenous ubiquitination. For this and all the subsequent ubiquitination assays, cell lysis was performed under strong denaturing conditions (2% SDS) to ensure that no proteins associated with PB2 could be coprecipitated, so that only PB2-specific ubiquitination through covalent linkages was detected. Antiubiquitin immunoblotting on the PB2 proteins precipitated with anti-Flag antibodies revealed a smear of PB2 polyubiquitination upon expression of PB2 alone, and coexpression of CRL4 factors enhanced this ubiquitination (Fig. 3A). Similar results were obtained when hemagglutinin (HA)-tagged ubiquitin was ectopically expressed together with PB2 and DDB1 or DCAFs (Fig. S4A). These results show that all three CRL4 factors are mediating the ubiquitination of PB2, suggesting that PB2 can be ubiquitinated by two CRL4-based E3 complexes, one containing DCAF12L1 and another containing DCAF11 as SRF, where DDB1 is a common adaptor. These E3 ligase complexes will be referred to as CRL4^D12L1^ and CRL4^D11^, respectively.

10.1128/mBio.00305-20.4 FIG S4 CRL4 E3 ligase complexes mediate PB2 ubiquitination *in vitro.* (A) HEK293T cells were transiently transfected with 3×Flag-PB2, Strep-DCAF11 (D11), Strep-DCAF12L1 (D12L1), or Strep-DDB1 together with HA-ubiquitin expression plasmids. At 36 h posttransfection, anti-Flag immunoprecipitation was performed from cell lysates, and ubiquitination of Flag-PB2 was analyzed by anti-HA immunoblotting. Expression of Flag-PB2 and Strep fusions was determined by immunoblotting assays in cell extracts as shown (Input). (B) HEK293 cells or HEK293 cells stably expressing Strep-DDB1, Strep-DCAF12L1, or Strep-DCAF11 were transfected with 3×Flag-PB2 expression plasmid. At 36 h posttransfection, cells were treated with MG132 (10 μM) for 4 h. PB2 ubiquitination was analyzed following anti-Flag immunoprecipitation and antiubiquitin immunoblotting. Download FIG S4, PDF file, 1.0 MB. Copyright © 2020 Karim et al. 2020 Karim et al. This content is distributed under the terms of the Creative Commons Attribution 4.0 International license.

The ubiquitination of PB2 when expressed alone is mediated by endogenous cellular E3 ubiquitin ligases. To analyze the involvement of the CRL4 E3 ligases in this baseline level of PB2 ubiquitination, we knocked down DDB1, DCAF12L1, or DCAF11 in HEK293 cells stably expressing their Strep-tagged version. The degree of PB2 ubiquitination measured in each cell line was similar to control HEK293 cells (Fig. S4B), indicating that stable expression of the Strep-CRL4 factors did not alter the endogenous levels of PB2 ubiquitination. PB2 polyubiquitination was reduced upon siRNA knockdown of DDB1 and the DCAFs (Fig. 3B). This reduction was detected even though the knockdown was only partial, especially for the DCAFs. We thus inferred that ubiquitination of PB2 is mediated, at least in part, by CRL4^D12L1^ and CRL4^D11^.

To further investigate whether CRL4 E3 ligases are mediating PB2 ubiquitination during IAV infection, we infected HEK293T cells with a recombinant H1N1_WSN_-PB2-Strep virus and prepared cell lysates at different times postinfection. Pulldown of Strep-PB2, followed by antiubiquitin immunoblotting, showed a robust polyubiquitination of PB2 during infection (Fig. 3C). The ubiquitination smear is detected as soon as the PB2 protein is detected during infection, and its intensity increases with the infection time. This suggests that ubiquitination of PB2 parallels its accumulation during the viral cycle. The ubiquitination of PB2 could also be detected in infected A549 cells (Fig. 3C), emphasizing that PB2 ubiquitination is not a cell-specific phenomenon. In addition, siRNA-mediated silencing of DDB1 or DCAFs reduced the level of PB2 ubiquitination in both HEK293T and A549 infected cells (Fig. 3D). Our results thus indicate that CRL4^D12L1^ and CRL4^D11^ are involved in the ubiquitination of PB2 during infection.

### CRL4^D12L1^ and CRL4^D11^ are mediating a nondegradative ubiquitination of PB2.

**FIG 4:**
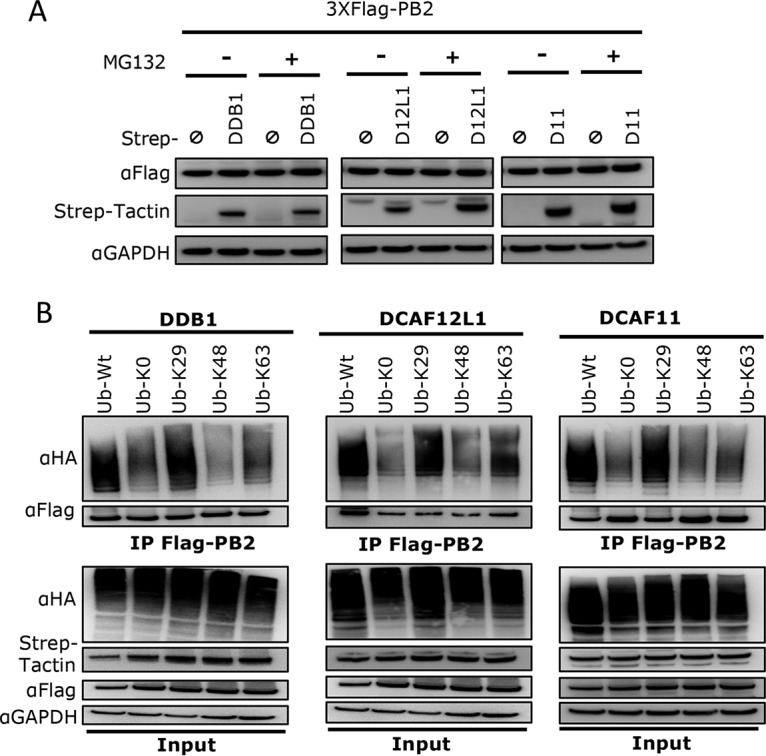
CRL4^D11^ and CRL4^D12L1^ mediate the nondegradative ubiquitination of PB2. (A) HEK293T cells were cotransfected with expression plasmids for 3×FLAG-PB2 and Strep-DDB1, Strep-DCAF12L1 (D12L1), Strep-DCAF11 (D11), or Strep-empty for 36 h and then treated with MG132 (+) for 4 h before lysis where indicated. Whole-cell lysates were prepared using Laemmli buffer and immunoblotted using indicated antibodies. (B) HEK293T cells were cotransfected with Strep-DDB1, Strep-DCAF12L1, or Strep-DCAF11 and with indicated HA-tagged ubiquitin mutants. At 36 h posttransfection, cells were lysed under strong denaturing conditions and subjected to Flag immunoprecipitation, followed by Western blotting with indicated antibodies.

Protein ubiquitination can have different consequences, one of the primary ones being induction of protein degradation. To examine if PB2 ubiquitination mediated by CRL4^D12L1^ and CRL4^D11^ affects the stability of PB2, HEK293T cells were cotransfected with Flag-tagged PB2 and Strep-tagged DDB1 or DCAFs for 24 h, in the presence or absence of MG132, and the steady-state level of PB2 was detected. The amount of PB2 in cell extracts did not differ whether or not DDB1 or DCAFs were ectopically expressed (Fig. 4A). MG132 treatment, while increasing the detection of endogenous ubiquitin linkage to PB2, did not induce overt modification of the steady-state PB2 levels (Fig. 4A). Similar results were obtained in A549 cells by ectopic expression of the DCAFs (Fig. S5), while DDB1 was not expressed at detectable levels.

10.1128/mBio.00305-20.5 FIG S5 CRL4 factors do not affect PB2 levels. A549 cells were cotransfected with expression plasmids for 3×Flag-PB2 and Strep-DCAF12L1 (D12L1), Strep-DCAF11 (D11), or Strep-empty for 24 h and then treated with MG132 for 4 h before lysis where indicated. Whole-cell lysates were prepared using Laemmli buffer and immunoblotted as indicated. The asterisk indicates an aspecific band. Download FIG S5, PDF file, 0.5 MB. Copyright © 2020 Karim et al. 2020 Karim et al. This content is distributed under the terms of the Creative Commons Attribution 4.0 International license.

Moreover, depletion of the CRL4 factors did not result in a rise in PB2 levels during infection, which would be expected if they cause PB2 degradation (Fig. 1B). In contrast, PB2 levels are decreased upon depletion of the CRL4 factors during infection, which is related to an effect on viral cycle progression. Altogether, these results indicate that ubiquitination of PB2 by CRL4^D12L1^ and CRL4^D11^ does not induce its degradation. Since nonproteolytic ubiquitination may regulate protein activity, we investigated whether the CRL4 factors could alter the transcription/replication activity of the viral polymerase. In an infectious context, the viral vRNA and mRNA copy number were not affected upon silencing of the CRL4 factors (Fig. S6A), suggesting that the viral transcription/replication was not impacted. These results were confirmed in a minireplicon assay, where a pseudogenome encoding the firefly luciferase is used as a reporter of viral transcription/replication. The knockdown of the CRL4 factors did not significantly alter the levels of firefly luciferase expressed by the minigenome reporter (Fig. S6B), suggesting that PB2 ubiquitination does not impact the transcription/replication activity of the polymerase.

10.1128/mBio.00305-20.6 FIG S6 Impact of CRL4 factors on PB2 transcription/replication activities. (A) Levels of vRNA and mRNA upon CRL4 knockdown. A549 cells were transfected with siRNA nontarget (NT) or siRNA targeting CRL4 factors for 48 h and infected with H1N1_WSN_ at an MOI of 3. At 6 h postinfection, total RNA was extracted using the RNeasy minikit (Qiagen). vRNAs and mRNAs from the NA segment were quantified by strand-specific RT-qPCR according to a protocol described in reference 34. The copy numbers are shown as the mean ± SD for three independent experiments in duplicate. (B) Minireplicon assays. HEK293T cells were transfected with siRNA nontarget (NT) or siRNA targeting CRL4 factors. Twenty-four hours later, cells were transfected with 25 ng of expression plasmids for PB1, PB2, and PA and 50 ng of NP plasmid of H1N1_WSN_ together with 10 ng of minigenome pPolI-Firefly and 5 ng of pTk-*Renilla* plasmids. At 24 h posttransfection, firefly luciferase and *Renilla* luciferase activities were measured using the Dual-Glo luciferase assay system (Promega). The results are expressed as percentages of NT and are shown as mean ± SD for four independent experiments in at least triplicates. Download FIG S6, PDF file, 0.1 MB. Copyright © 2020 Karim et al. 2020 Karim et al. This content is distributed under the terms of the Creative Commons Attribution 4.0 International license.

### CRL4^D12L1^ and CRL4^D11^ are mediating K29-linked ubiquitination of PB2.

The type of ubiquitin chain linked to a protein determines its fate. Especially, K48-linked polyubiquitination is responsible for protein targeting to the proteasome for degradation, whereas K63-linked ubiquitination is the most common nondegradative ubiquitination. To explore the type of ubiquitin chains attached to PB2 by the CRL4 factors, we used expression vectors for HA-tagged ubiquitin mutants where a single lysine residue is conserved while all other lysines are mutated to arginine. As shown in Fig. 4B, PB2 ubiquitination could be detected with K29 ubiquitin mutant at similar levels as with the wild-type (wt) ubiquitin, when coexpressed with CRL4 factors. K29-linked ubiquitination thus appears to be the major type of PB2 ubiquitination mediated by CRL4^D12L1^ and CRL4^D11^ complexes. Some degree of K63-linked ubiquitination of PB2 could be detected as well, although to lower levels than the wild-type or K29-linked ubiquitination.

To confirm these results, we used the reverse ubiquitin mutants, where only one lysine residue, K29, K48, or K63, is mutated to arginine (Fig. S7). The CRL4 factor-mediated ubiquitination of PB2 was detected upon expression of the ubiquitin K48R mutant to levels similar to wt ubiquitin, firmly establishing that the CRL4 E3 ligases do not mediate K48-linked ubiquitination of PB2. This is in line with our results showing that PB2 ubiquitination does not induce its proteasome-mediated degradation. The level of PB2 ubiquitination was consistently lower upon expression of Ub-K29R, while a partial effect was observed using Ub-K63R (Fig. S7). All in all, our data indicate that CRL4^D12L1^ and CRL4^D11^ are catalyzing a nonproteolytic ubiquitination of PB2 predominantly composed of K29-linked and possibly of K29-K63 mixed polyubiquitin chains.

10.1128/mBio.00305-20.7 FIG S7 CRL4 mediates nondegradative ubiquitination of PB2. HEK293T cells were cotransfected with expression plasmids for Strep-DDB1, Strep-DC1F12L1, or Strep-DCAF11 together with 3×Flag-PB2 and indicated ubiquitin mutants fused with MYC-tag. At 36 h posttransfection, cells were subjected to Flag pulldown and analyzed by Western blotting with indicated antibodies. Download FIG S7, PDF file, 1.5 MB. Copyright © 2020 Karim et al. 2020 Karim et al. This content is distributed under the terms of the Creative Commons Attribution 4.0 International license.

### CRL4s mediate different patterns of PB2 C-terminal domain ubiquitination.

**FIG 5:**
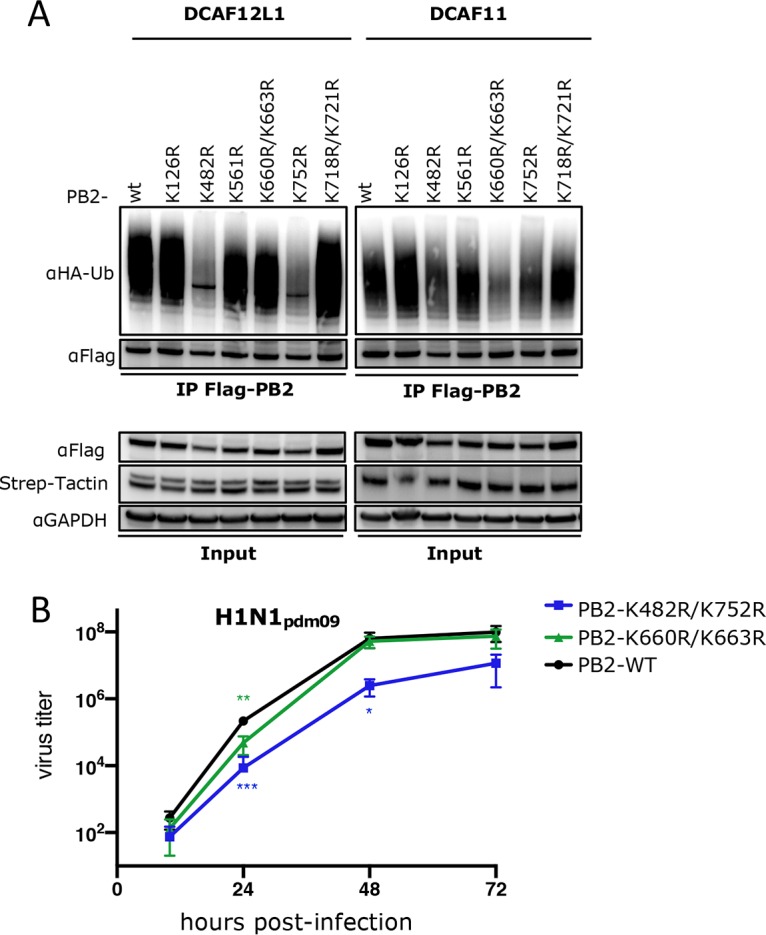
CRL4s mediate different patterns of PB2 ubiquitination that are involved in infection. (A) HEK293 cells stably expressing Strep-DCAF12L1 or Strep-DCAF11 were cotransfected with the indicated PB2 mutants fused with 3×Flag tag together with HA-ubiquitin. At 48 h posttransfection, cell lysates were subjected to Flag pulldown and analyzed by immunoblotting with the indicated antibodies. Expression of Flag-PB2 and Strep fusions was monitored in cell lysate (Input). (B) A549 cells infected at an MOI of 0.001 with recombinant H1N1_pdm09_ viruses, either wild type or mutated in the PB2 lysines targeted by each of the CRL4 E3 ligases as indicated. Viral titers were determined in triplicates by plaque-forming assay at the indicated time points, and significance was measured using one-way ANOVA (ns, *P* > 0.05; *, *P* ≤ 0.05; **, *P* ≤ 0.01; ***, *P* ≤ 0.001).

Substrate proteins often contain multiple receptor lysine residues for ubiquitination, leading to a complex pattern of ubiquitination. To unravel the ubiquitination pattern of PB2, we analyzed the CRL4-mediated ubiquitination of the PB2-Nt and PB2-Ct domains separately as done for full-length PB2. PB2-Ct was ubiquitinated at a level similar to that of the full-length protein, while marginal ubiquitination of PB2-Nt was detected (Fig. S8). This suggested that the PB2-Ct contains the major sites of CRL4^D12L1^- and CRL4^D11^-mediated ubiquitination. In an attempt to further delineate the lysine residues responsible for this ubiquitination in PB2, we used the UbiSite server (http://csb.cse.yzu.edu.tw/UbiSite/index.php) to predict ubiquitination sites in the PB2 protein of H1N1_WSN_, H1N1_pdm09_, and H3N2. The same eight lysine residues, conserved in the three strains, scored with high confidence as predicted ubiquitination sites. They were located at the surface of PB2 in the crystal structure, and all except one were located in the C-terminal domain of PB2. We mutated the 8 high-confidence lysines to arginine, either alone or in combination when they were in close proximity in the viral polymerase structure, and assessed the ubiquitination of the lysine-mutated PB2 proteins in cells stably expressing DCAF12L1 or DCAF11. Mutation of lysine K482 or K752 strongly reduced PB2 ubiquitination upon expression of DCAF12L1, suggesting that these two lysines are the main targets for ubiquitination by CRL4^D12L1^ (Fig. 5A). These two mutated PB2 proteins, however, are still ubiquitinated upon expression of DCAF11, to levels roughly similar to that of the wild-type protein. The effect of lysine mutations is less pronounced under DCAF11 expression, with mutations KK660/663RR having only a moderate effect on the level of PB2 ubiquitination (Fig. 5A). These results suggest that CRL4^D11^-mediated PB2 ubiquitination likely involves multiple lysines in the C-terminal two-thirds of PB2. Altogether, our data underscore that the CRL4^D12L1^ and CRL4^D11^ ligases are mediating different patterns of ubiquitination on PB2.

10.1128/mBio.00305-20.8 FIG S8 CRL4 factors ubiquitinate the C-terminal domain of PB2. HEK293T cells were transiently transfected with expression plasmids for Strep-DDB1, Strep-DCAF12L1, or Strep-DCAF11 together with 3×Flag-PB2 full-length (FL), N-terminal (Nt), or C-terminal (Ct) domains of PB2. At 36 h posttransfection, cells were treated with MG132 (10 μM) for 4 h. Anti-Flag immunoprecipitation was performed, and results were analyzed by Western blotting with indicated antibodies. Expression of Flag-PB2 and Strep fusions was monitored in cell lysate (Input) using the indicated antibodies. Download FIG S8, PDF file, 0.9 MB. Copyright © 2020 Karim et al. 2020 Karim et al. This content is distributed under the terms of the Creative Commons Attribution 4.0 International license.

### Functional involvement of CRL4s mediates PB2 ubiquitination.

We studied the effect of CRL4-targeted lysine mutation on H1N1_pdm09_ polymerase activity using a minireplicon assay. Mutation of K660R/K663R had no impact on viral polymerase activity. Surprisingly, K482R and K752R mutations enhanced viral polymerase activity by 2- to 2.5-fold (Fig. S9). However, this effect is likely unrelated to an impaired CRL4^D12L1^-mediated PB2 ubiquitination, since the silencing of the CRL4 factors does not affect viral transcription/replication, neither during infection nor in a minireplicon assay (Fig. S6).

10.1128/mBio.00305-20.9 FIG S9 Polymerase activity of PB2 mutated in the CRL4-targeted lysines. HEK293T cells were transfected with 25 ng of expression plasmids for PB2 wild type or mutated for lysine residues as indicated, PB1, and PA and 50 ng of NP plasmid of H1N1_pdm09_, together with 10 ng of minigenome pPolI-Firefly and 5 ng of pTk-*Renilla* plasmids. At 24 h posttransfection, firefly luciferase and *Renilla* luciferase activities were measured using the Dual-Glo luciferase assay system (Promega). The results are expressed as percentages of NT and are shown as mean ± SD for three independent experiments in at least triplicates. Download FIG S9, PDF file, 0.02 MB. Copyright © 2020 Karim et al. 2020 Karim et al. This content is distributed under the terms of the Creative Commons Attribution 4.0 International license.

To address the functional importance of CRL4-mediated ubiquitination of PB2 in IAV infection, we constructed recombinant H1N1_pdm09_ virus containing PB2 K482R/K752R or PB2 K660R/K663R mutations by reverse genetics. The replication kinetics of these mutant viruses was then studied in A549 cells (Fig. 5B). The H1N1_pdm09_ PB2 K660R/K663R mutant was slightly attenuated 24 h postinfection and recovered normal levels of viral production at later time points. We hypothesize that, as CRL4^D11^ was shown to target multiple lysines of PB2, mutating the 660 and 663 lysine residues alone is not sufficient to observe a strong effect. In contrast, the H1N1_pdm09_ PB2 K482R/K752R mutant displayed a more pronounced and consistent attenuated phenotype over time. The mutation of these lysines abrogates CRL4^D12L1^-mediated ubiquitination of PB2, suggesting that the lack of CRL4^D12L1^-mediated ubiquitination of PB2 impairs viral production. Overall, our results support that ubiquitination of PB2 by the CRL4 E3 ligases is contributing to IAV infection.

## DISCUSSION

Post-translational modification of viral proteins is an important feature driving their multifunctional nature. Surprisingly little information is available on PTMs that regulate the PB2 subunit of the influenza A virus polymerase complex, and ubiquitination is one of the few PTMs that have been documented (15, 17). In our earlier work, we provided evidence for pairwise interactions between the PB2 protein of several influenza A virus strains and three components of CRL4 E3 complexes, the adaptor DDB1 and two SRFs, DCAF12L1 and DCAF11 (18). In addition, DDB1 protein has been detected as an associated factor of the vRNPs by mass spectrometry (MS) and co-IP experiments (21). We show here that PB2 associates with the adaptor-SRF interacting pairs typical of CRL4 E3 ligase complexes, DDB1-DCAF12L1 and DDB1-DCAF11. This led us to conclude that PB2 associates with two CRL4s, containing either DCAF12L1 or DCAF11 as SRF (CRL4^D12L1^ and CRL4^D11^, respectively). The interaction of PB2 with the CRL4 factors is detected in an infectious context, and silencing of either the adaptor (DDB1) or the SRF (DCAF12L1 or DCAF11) components reduces the ubiquitination level of PB2. These results indicate that the CRL4^D12L1^ and CRL4^D11^ E3 ligases are mediating PB2 ubiquitination during infection. Our results also imply that PB2 is recruited to the CRL4^D12L1^ and CRL4^D11^ E3 ligases through a 2-fold binding, to both the adaptor and the SRF. Such bimodal interaction is an atypical mode of substrate protein recruitment to CRL E3 ligases, which differs from the canonical one consisting in sequential adaptor-SRF and SRF-substrate interactions. A number of viruses, such as hepatitis B virus (HBV), simian virus 5 (SV5), simian immunodeficiency virus (SIV), human papillomavirus (HPV), human immunodeficiency virus type 1 (HIV-1), herpes simplex virus (HSV), and some paramyxoviruses, have been documented to hijack CRL E3 ligases, through interactions either with the DDB1 adaptor or with SRFs, but no bimodal interaction has been documented (22).

Ubiquitination of PB2, depending on its prior ADP-ribosylation, was shown to induce proteasome-mediated degradation of the protein (15). In another study, the ubiquitination of PB2 was reported, together with that of the other replication proteins PB1, PA, and NP, and shown to play a proviral role independent of their degradation, but the ubiquitination process was not further characterized (17). In this work, we provide several pieces of evidence that both CRL4^D12L1^ and CRL4^D11^ mediate a nonproteolytic ubiquitination of PB2. First, the PB2 protein levels are not increased upon depletion of the CRL4 components, which would be expected if the CRL targeted PB2 for proteasomally mediated degradation. Second, CRL4s are not mediating the addition of K48-linked polyubiquitin chains, which are the principal signals for protein targeting to proteasome-mediated degradation. We were surprised that binding of PB2 to two types of CRL4s led to its ubiquitination, because it indicates a redundancy between two closely related E3 ligases for a single substrate protein ubiquitination. However, DCAF12L1 and DCAF11 do not complement each other functionally in the context of IAV infection, since the siRNA-mediated depletion of each of them individually affects the viral cycle.

Indeed, our previous work indicated that virus production was decreased upon silencing of the CRL4 factors (18). In contrast, ectopic expression of DDB1 and DCAF11 increased viral replication, and a similar trend was observed for DCAF12L1 (18). We show here that the depletion of the CRL4 factors results in a hampered viral cycle, reflected by a slowed-down accumulation of viral proteins and a delayed vRNP export. It is indicative of suboptimal completion of viral processes, which ultimately results in a reduced viral production. The activities of CRL4^D12L1^ and CRL4^D11^ thus favor the viral replication cycle. Virus replication kinetics showed a milder effect of CRL4 silencing for the lab-adapted H1N1_WSN_ strain than for the seasonal strains H1N1_pdm09_ and H3N2. This does not seem to be due to a lower binding of PB2 from H1N1_WSN_ to the CRL4 factors, because the signals in the GPCA, which are good indicators of binding affinities, were not the lowest (18, 23). We assume that since the H1N1_WSN_ virus is well adapted to growth in cell culture, it is potentially less affected by depletion of particular cell factors.

Our results also indicate that the CRL4^D12L1^ and CRL4^D11^ E3 ligases induce different patterns of lysine ubiquitination on PB2, supporting the differential involvement of these CRL4s in infection. Recombinant H1N1_pdm09_ viruses insensitive to CRL4^12L1^-mediated ubiquitination through mutation of PB2 in K482/K752 lysines showed an attenuated phenotype. These results suggest that the proviral effect of the CRL4^D12L1^ is mediated, at least in part, by ubiquitination of PB2. Regarding CRL4^D11^, mutation of PB2 in K660/K663 transiently affected viral production. This may be because multiple lysines are targeted by CRL4^D11^, and therefore, this PB2 mutant might not be fully resistant to CRL4^D11^-mediated ubiquitination. Our results nevertheless support the functional involvement of PB2 ubiquitination by CRL4 E3 ligases in IAV infection.

The CRL4 components can directly bind to the PB2 protein either in isolation or when complexed with PB1 and PA in the trimeric polymerase complex. Pulldown analyses in the context of infection suggest that the CRL4s can associate with vRNPs, since the other vRNP constituents PB1, PA, and NP are copurified (not shown). The vRNPs are the templates for viral RNA transcription and replication driven by the viral polymerase. No significant effect of CRL4 silencing on the viral transcription/replication has been detected, neither in an infectious context nor in a minireplicon assay. However, mutation of CRL4^D12L1^-targeted PB2 lysines K482R and K752R increased polymerase activity, which is inconsistent with the attenuated phenotype of the PB2-K482R/K752R H1N1_pdm09_. We therefore concluded that the K482R and K752R mutations seem to have effects in the minigenome assay that are not relevant in infection. In a previous work, the silencing of DDB1 was shown to reduce the polymerase activity of H5N1, but the effect was less significant for H1N1_WSN_ (24). It may be assumed that the reason why no posttranslational modifications activating PB2 have been detected so far is that they do not primarily regulate the activities of the protein involved in viral transcription/replication. The CRL4-mediated PB2 ubiquitination is likely involved in other activities of PB2, which are poorly known. It might also regulate the nuclear export of the protein in the course of the viral cycle, which is in line with the observed effect of CRL4 factor silencing. We speculate that the different nondegradative PB2 ubiquitination patterns could be involved in the proper orchestration of discrete viral processes remaining to be characterized.

The polyubiquitination of PB2 by the CRL4s studied here mainly consists in K29 linkages of Ub chains. The K29 linkage is the most abundant atypical ubiquitin linkage in unstressed cells (25, 26). The studies on K29-linked protein ubiquitination mostly report proteasome-independent functions, such as Wnt signaling regulation (27), antiviral innate immune response (28), or protein aggregation in the context of Parkinson disease (29). We show here that the K29-linked ubiquitination of PB2 is a nondegradative ubiquitin signal, further supporting proteasome-independent functions of this linkage type. However, the amount of K29 poly-Ub increases following proteasomal inhibition (25), which could explain why endogenous ubiquitination of PB2 is better detected upon MG132 treatment. Some degree of K63-linked ubiquitination could also be detected, suggesting the combined existence of K29 and K63 linkages on ubiquitinated PB2. This is evocative of mixed or heterotypic K29-K63 polyubiquitination of PB2, in line with the fact that K29-linked chains have been shown to exist primarily within mixed ubiquitin chains (25). Compared to the typical K48 and K63 ubiquitin signals, the cellular roles of the atypical linkages are less clear. They nevertheless emerge as important regulators of cell homeostasis (4). This is to our knowledge the first evidence of a virus exploitation of K29 ubiquitin linkage, providing a pioneering illustration of the role of this atypical linkage in the regulation of pathogens.

## MATERIALS AND METHODS

### Cell culture and stable cell lines.

HEK293T and A549 cells were grown in Dulbecco’s modified Eagle’s medium supplemented with 10% fetal calf serum (FCS). MDCK cells were grown in modified Eagle’s medium supplemented with 5% FCS. For the generation of stable cell lines, HEK293 cells were transfected with expression vectors for Strep tag DDB1, DCAF12L1, DCAF11, or mCherry and then selected by neomycin treatment. Single cell clones were sorted, expanded, and assessed for Strep fusion expression levels.

### Plasmids.

The open reading frames (ORFs) encoding virus-related proteins were derived from influenza A/WSN/33 virus. The Gateway entry plasmids containing the DDB1, DCAF12L1, and DCAF11 ORFs were obtained from the human ORFeome resource (Center for Cancer Systems Biology [CCSB] human ORFeome collection). They were transferred into Gluc1, Gluc2, GlucFL, 3×Flag, and Strep fusion-expressing vectors using the Gateway technology.

### Antibodies.

For Western blotting assays, the membranes were incubated with primary antibodies directed against PB2 (GeneTex), NP (Kerafast), polyclonal anti-A/PR/8/34 virions (30), NS1 (kindly provided by Daniel Marc, INRA-Tours, France), NA (GeneTex), HA (GeneTex), *Gaussia* luciferase (New England Biolabs), DDB1 (Sigma), Flag (Sigma), HA tag (C29F4; Cell Signaling), Myc (kindly provided by Amel Mettouchi and Emmanuel Lemichez, Institut Pasteur Paris), ubiquitin (P4D1; Invitrogen), and glyceraldehyde-3-phosphate dehydrogenase (GAPDH) (Pierce) and peroxidase-conjugated secondary antibodies (GE Healthcare) or peroxidase-conjugated Strep-Tactin (IBA).

### siRNA transfection and efficiency.

Small interfering RNAs (siRNAs) targeting DDB1, DCAF12L1, and DCAF11 were purchased from Sigma (ON-TARGETplus individual siRNAs; Sigma). Nontarget siRNA (ON-TARGETplus nontargeting control pool; Dharmacon) was used as a negative control. A549 cells were transfected with 25 nM siRNA using the Interferin transfection reagent (Polyplus).

### Viruses and infection.

For multicycle growth assays, cells were infected with H1N1_WSN_, H1N1_pdm09_, or H3N2, and the production of infectious virus particles in the culture supernatant was determined using a plaque assay on MDCK-SIAT cells as described in reference 31. For single-cycle infection assays, cells were infected with either H1N1_WSN_ or H1N1_pdm09_ at an MOI of 3 to 5 PFU/cell. The influenza virus strain A/WSN/33 (H1N1_WSN_) is described in reference 32. A549-adapted A/Centre/1003/2012 (H3N2) and A/Bretagne/7608/2009 (H1N1_pdm09_) viruses were described in reference 18. Recombinant H1N1_pdm09_ viruses mutated in lysines of PB2 were produced by reverse genetics as described previously (32). Recombinant H1N1_WSN_ encoding PB2-Strep has been described in reference 33.

### Protein complementation assays to detect multiprotein complexes.

The protein complementation assay was performed as described in reference 19. Briefly, HEK293T cells were cotransfected with the expression plasmids encoding a polymerase subunit (PB2) fused to Gluc2 and DCAF11 or DCAF12L1 fused with Gluc1 together with a Strep-tagged DDB1 or Strep-empty expression plasmid. At 24 h posttransfection, cells were lysed using *Renilla* lysis buffer (Promega), and Strep-Tactin pulldown was performed. Luciferase activity was measured in 1/10 of cell lysates before pulldown and in the Strep-Tactin bead eluates. The luciferase activity retained on the beads was calculated as the percentage of luciferase activity before pulldown and compared for Strep-DDB1-loaded beads to Strep-empty beads. For the detection of quaternary complexes, HEK293T cells were cotransfected with the expression plasmids encoding the viral polymerase proteins PA fused to Gluc1 and PB1 fused to Gluc2, together with 3×Flag-tagged PB2 or 3×Flag-empty plasmid and Strep-tagged DDB1, Strep-tagged DCAF12L1, Strep-tagged DCAF11, or Strep-empty plasmid. The retention of the protein complex was calculated as described above.

### Strep pulldown and immunoprecipitation.

HEK293T cells were transiently cotransfected with the indicated expression plasmids for 3×Flag and Strep tag fusion proteins. At 24 h posttransfection, cells were lysed in 20 mM morpholinepropanesulfonic acid (MOPS)-KOH (pH 7.4), 120 mM KCl, 2 mM beta-mercaptoethanol, 0.5% IGEPAL. Cell lysates were incubated with Strep-Tactin beads (Strep-Tactin Sepharose high performance; GE Healthcare) for 2 h and washed 3 times in lysis buffer. Protein complexes were eluted from Strep-Tactin beads with desthiobiotin (IBA) and diluted in Laemmli buffer (Invitrogen) for Western blot analysis. For immunoprecipitation, cell lysates were incubated with anti-PB2 or control IgG antibody together with protein A Dynabeads (Invitrogen) for 2 h, beads were washed, and then precipitated proteins were analyzed by Western blotting.

### *In vitro* ubiquitination assay.

For endogenous ubiquitination detection, transfected HEK293T cells or infected cells were treated with MG132 for 4 h prior to lysis. Cells were lysed in 2% SDS for 10 min and then diluted in 50 mM Tris-HCl (pH 7.4), 150 mM NaCl, 2 mM EDTA, 5 mM dithiothreitol (DTT), 1% NP-40, and 0.5% deoxycholate (DOC). The PB2 protein was precipitated from the clarified supernatants by Strep pulldown or anti-Flag immunoprecipitation. Ubiquitinated PB2 was detected by Western blotting using antiubiquitin antibodies. For detection of ubiquitination under ectopic expression of HA-tagged or Myc-tagged ubiquitin, cell lysates were prepared without treatment with MG132. PB2 was immunoprecipitated with anti-Flag antibodies, and its ubiquitinated forms were detected by Western blotting using anti-HA or anti-Myc antibodies.

### Immunofluorescence.

A549 cells were fixed with phosphate-buffered saline (PBS)–4% paraformaldehyde for 20 min, permeabilized with PBS–0.1% Triton X-100 for 10 min, and incubated with anti-NP (AA5H; Abcam; 1/500) or anti-PB2 (1/200) antibodies and then with DyeLight488 anti-mouse or DyeLight533 anti-rabbit antibodies (1/500; Invitrogen) together with Hoechst 33342 (Invitrogen; diluted to 1 μg/ml). The samples were mounted in ProLong Gold mounting medium (Invitrogen).

### Statistics.

Statistical analyses were performed with GraphPad Prism (GraphPad Software Inc.) using ordinary one-way analysis of variance (ANOVA) with Dunnett’s multiple-comparison test or two-tailed unpaired Student’s *t* test.
