# Supplementary figures and images for: Nonproteolytic K29-Linked Ubiquitination of the PB2 Replication Protein of Influenza A Viruses by Proviral Cullin 4-Based E3 Ligases
Source: mBio. 2020 Apr 7;11(2):e00305-20. doi: 10.1128/mBio.00305-20 (PMC7157767; doi:10.1128/mBio.00305-20)

A

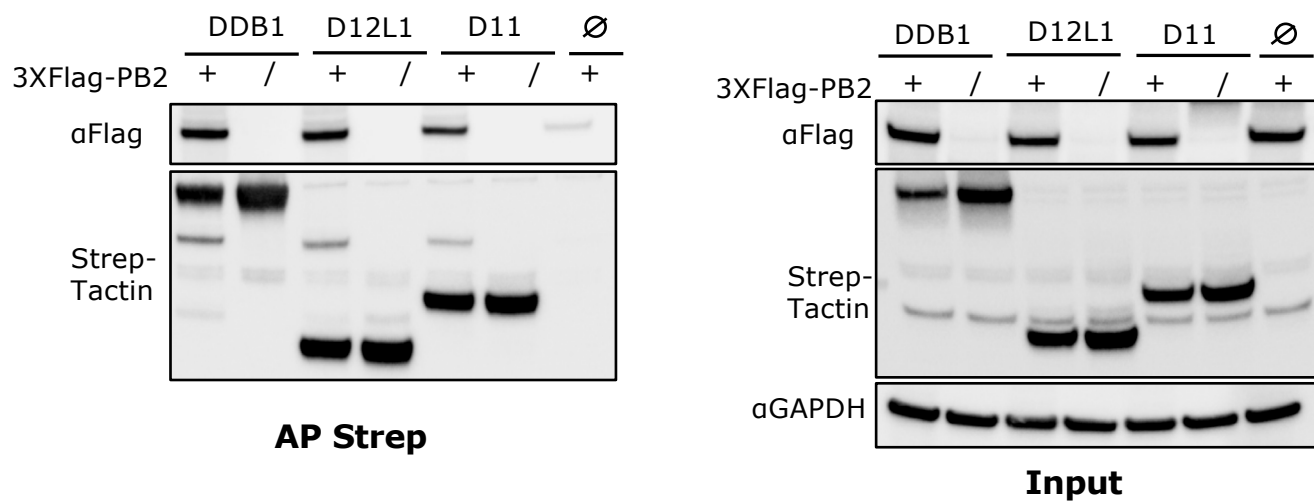

B

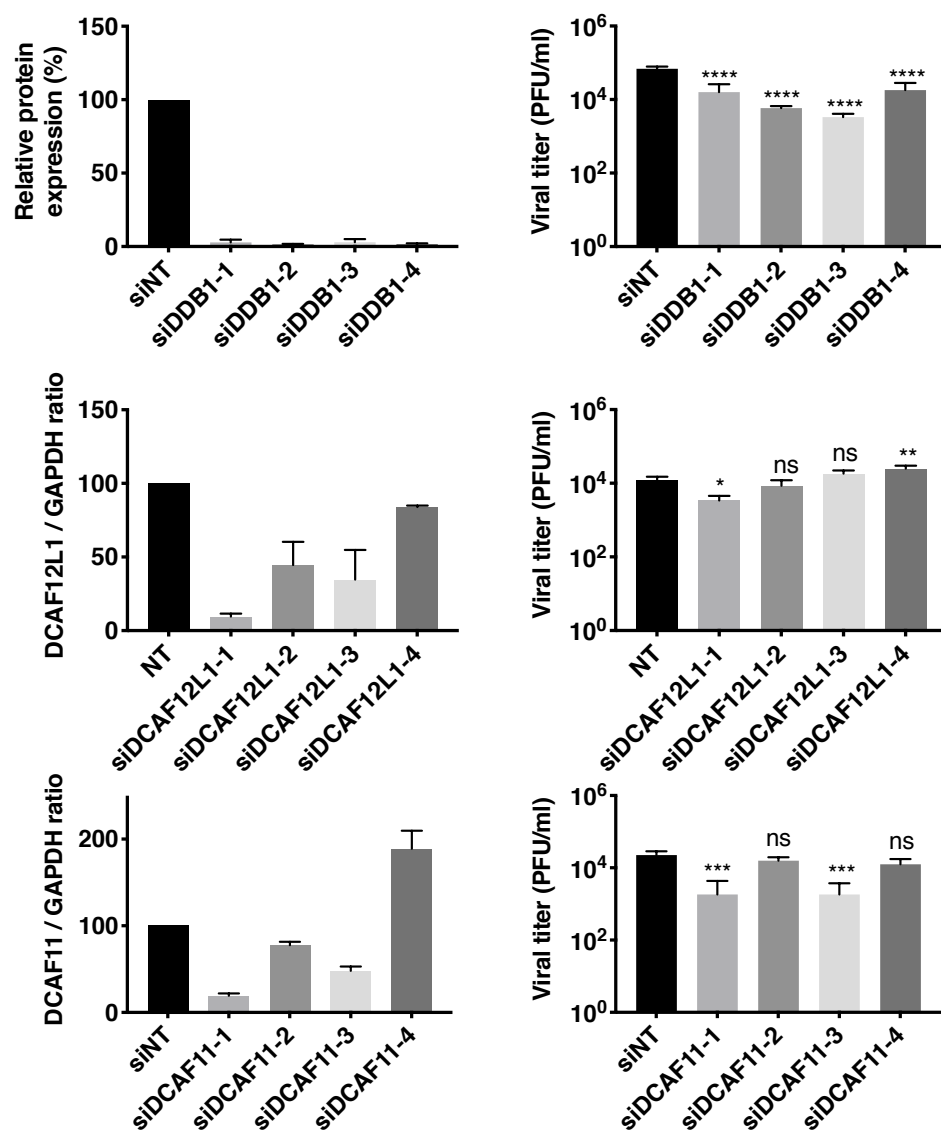

Supplement: FIG S1 [file mBio.00305-20-sf001.pdf]

A

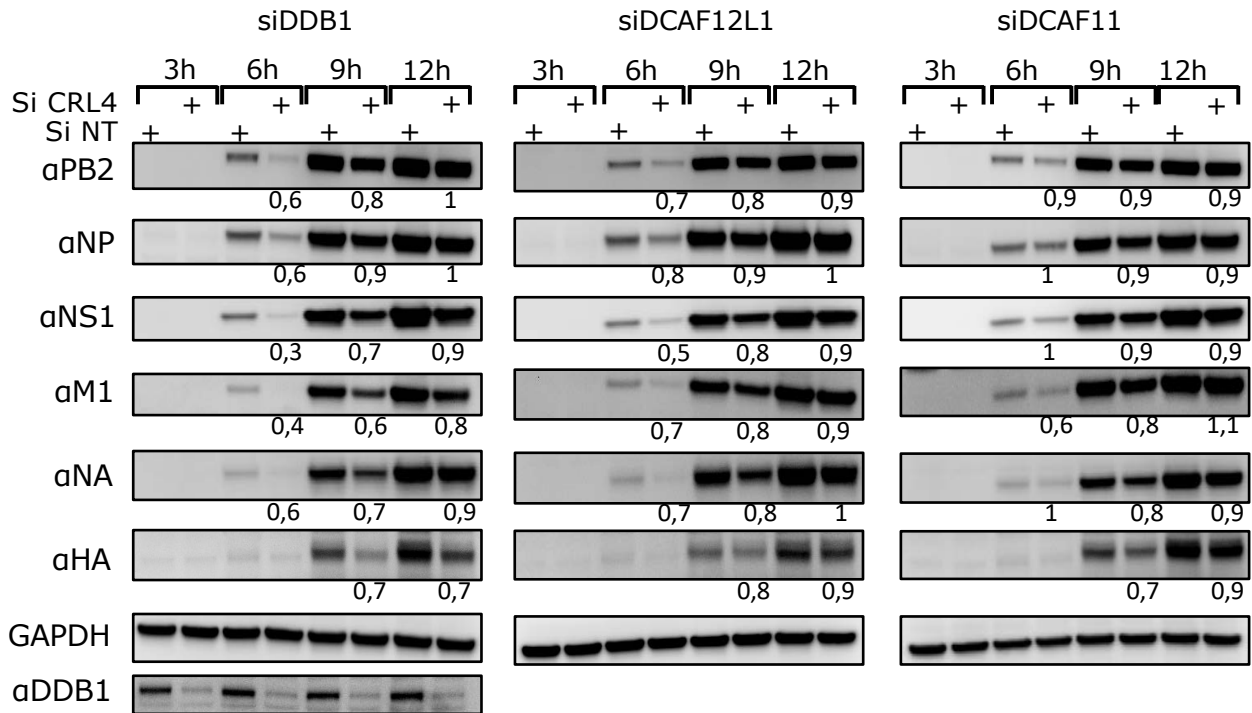

B

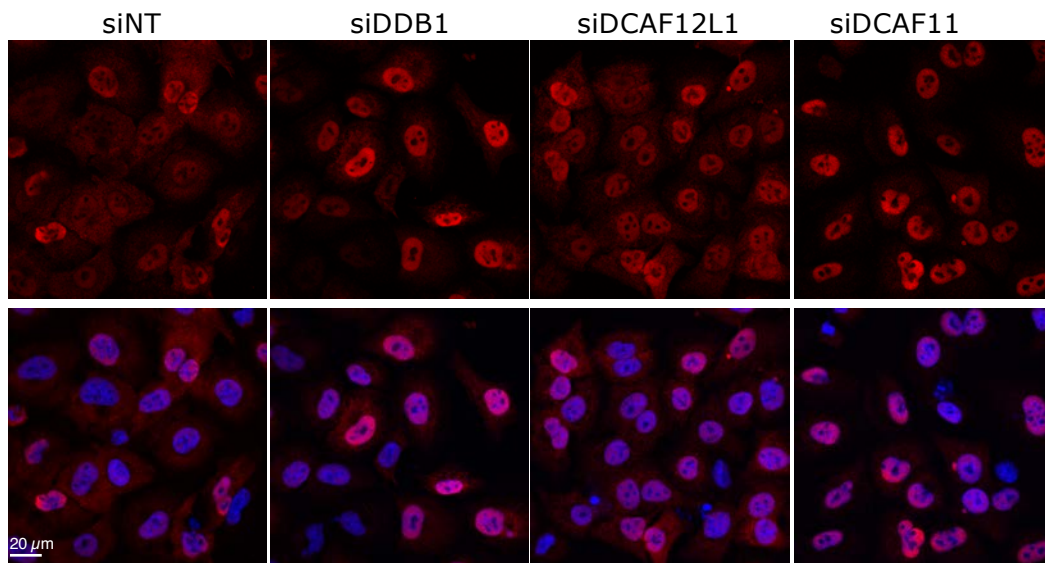

C

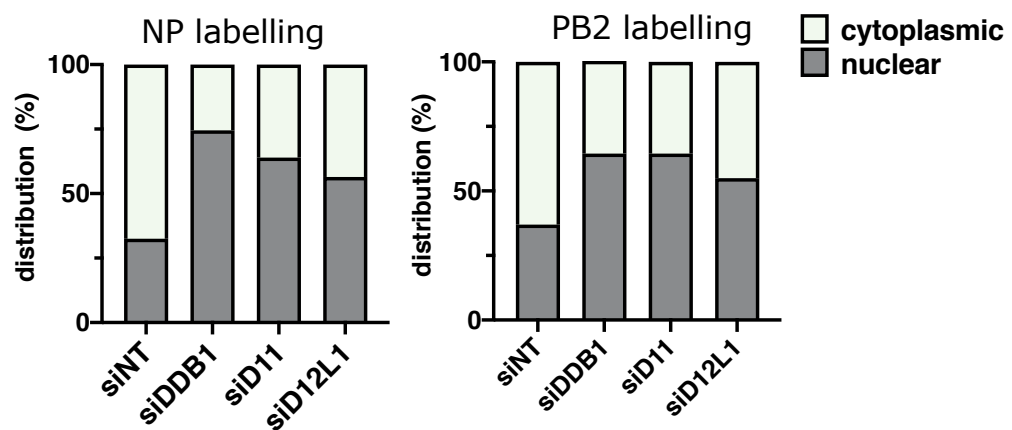

Supplement: FIG S2 [file mBio.00305-20-sf002.pdf]

A

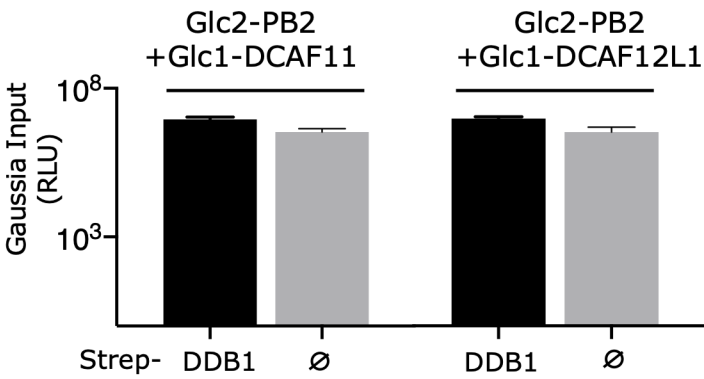

B

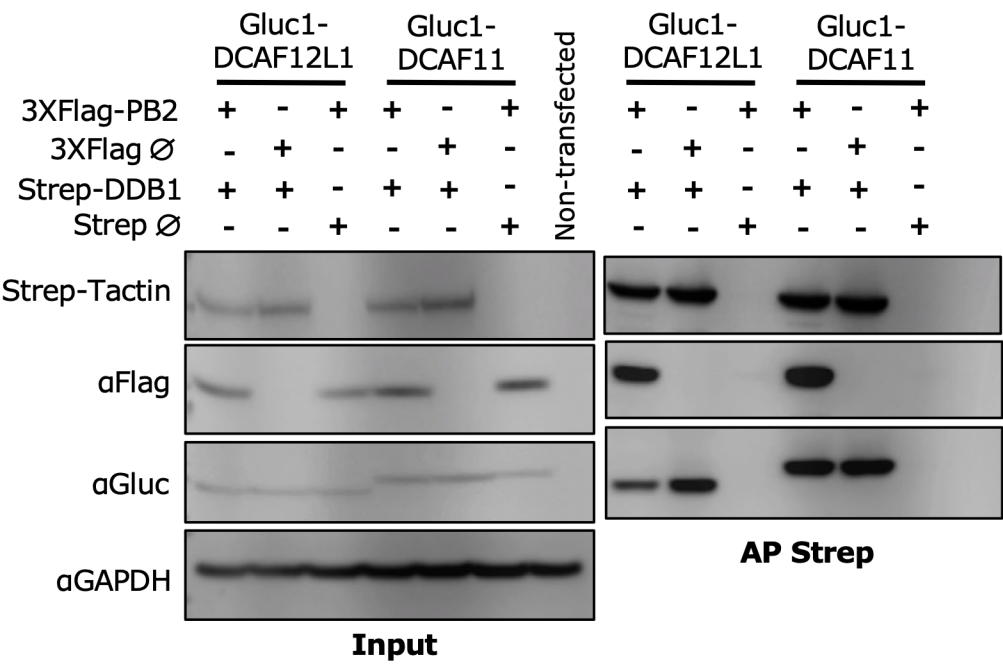

C

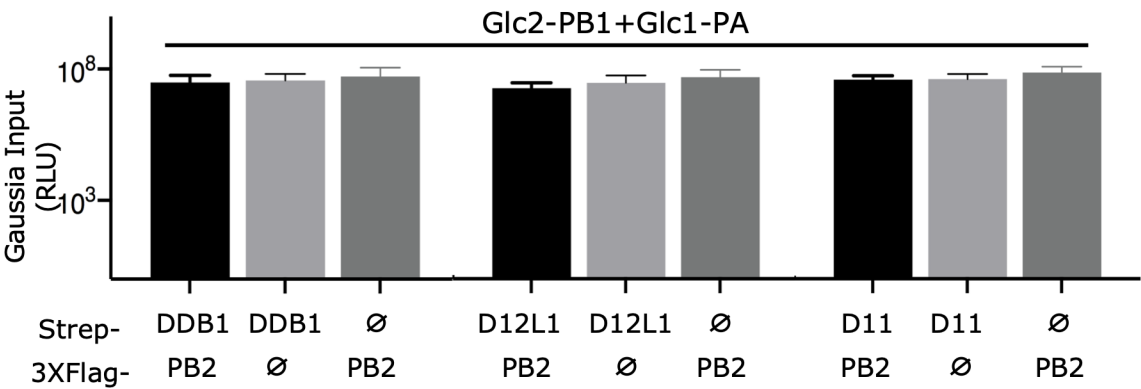

Supplement: FIG S3 [file mBio.00305-20-sf003.pdf]

**A**

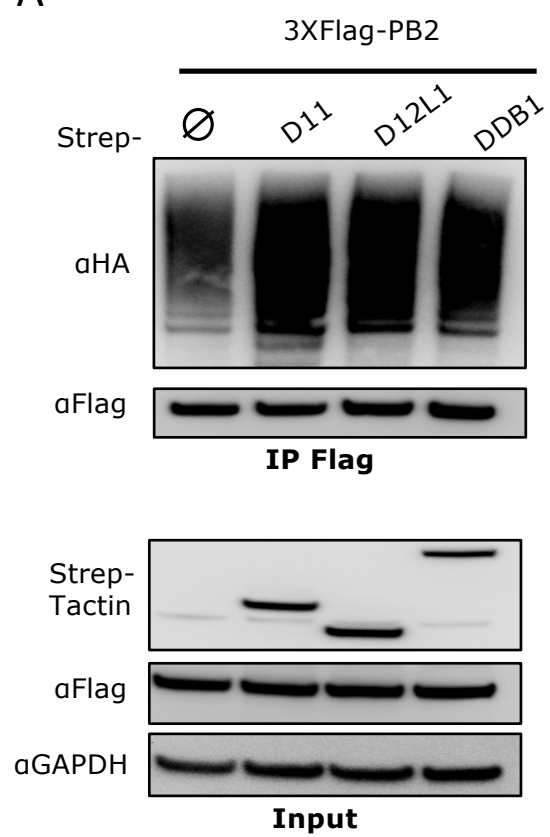

**B**

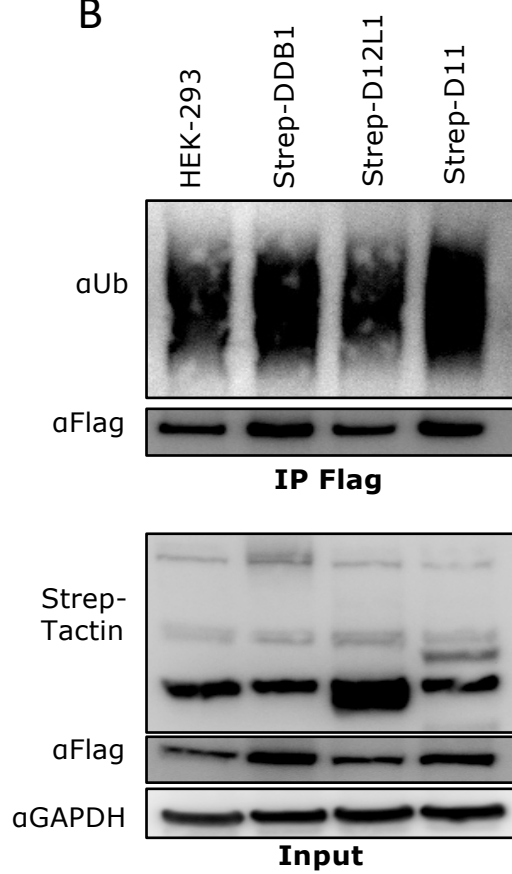

Supplement: FIG S4 [file mBio.00305-20-sf004.pdf]

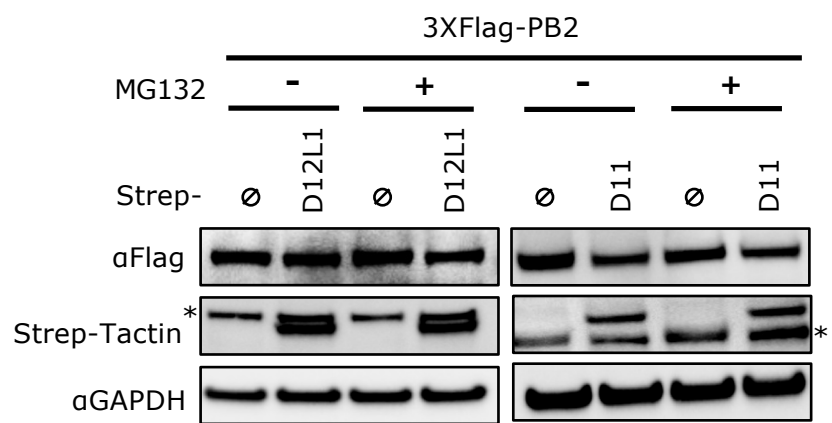

Supplement: FIG S5 [file mBio.00305-20-sf005.pdf]

A

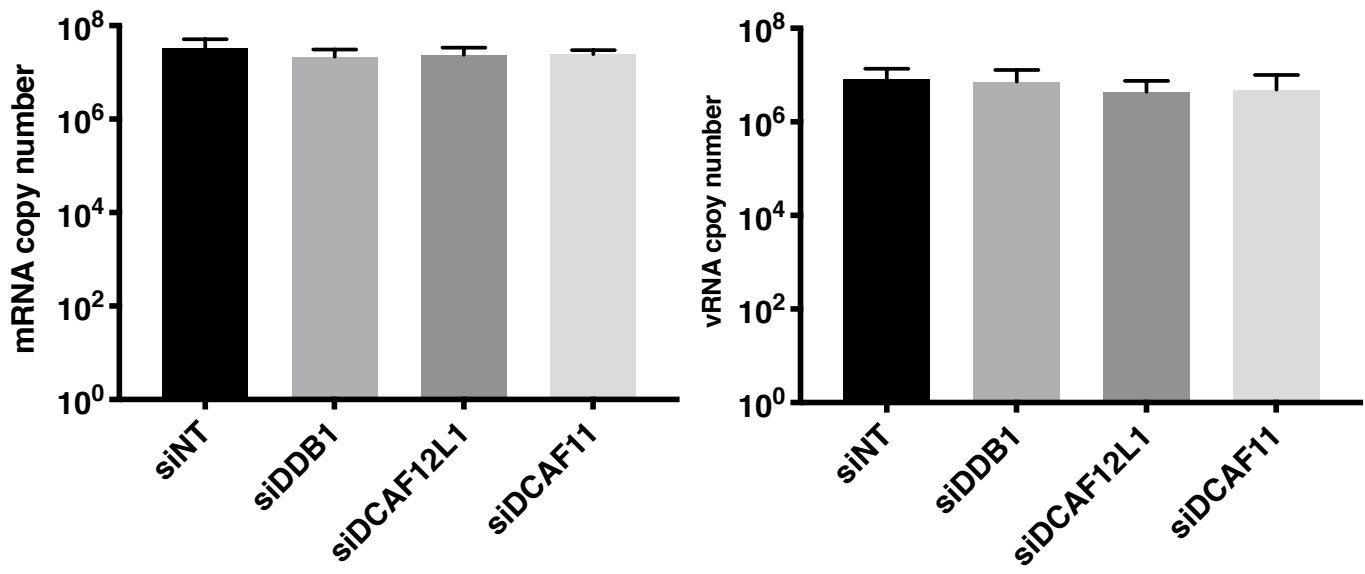

B

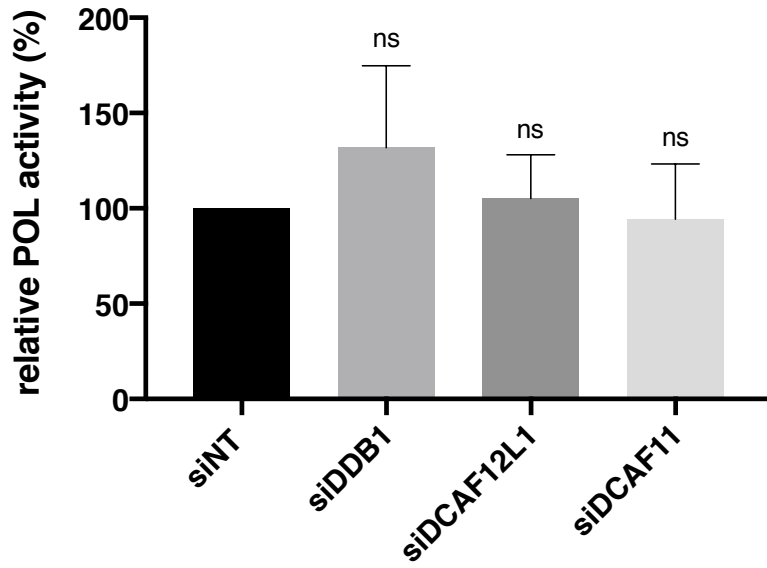

Supplement: FIG S6 [file mBio.00305-20-sf006.pdf]

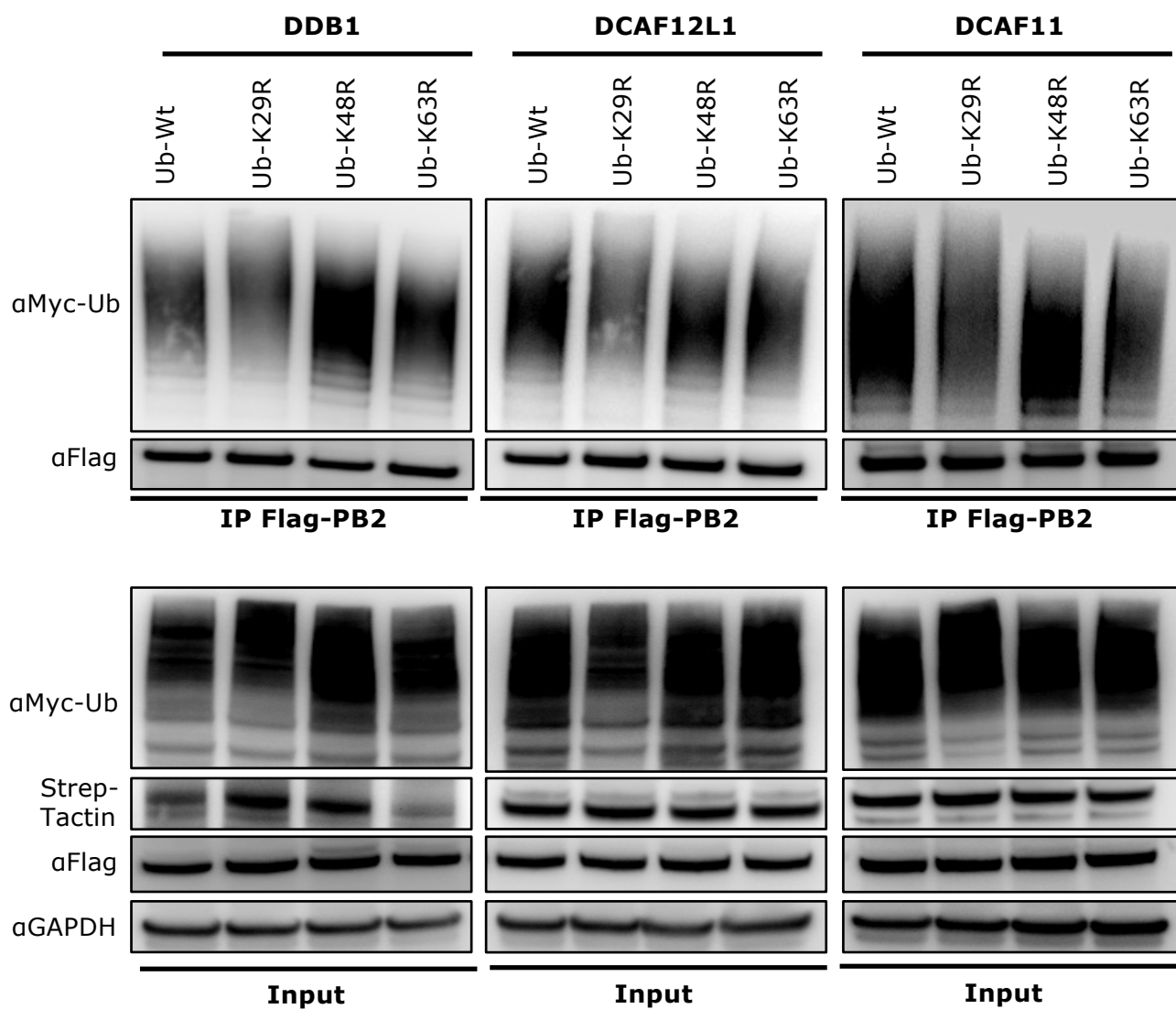

Supplement: FIG S7 [file mBio.00305-20-sf007.pdf]

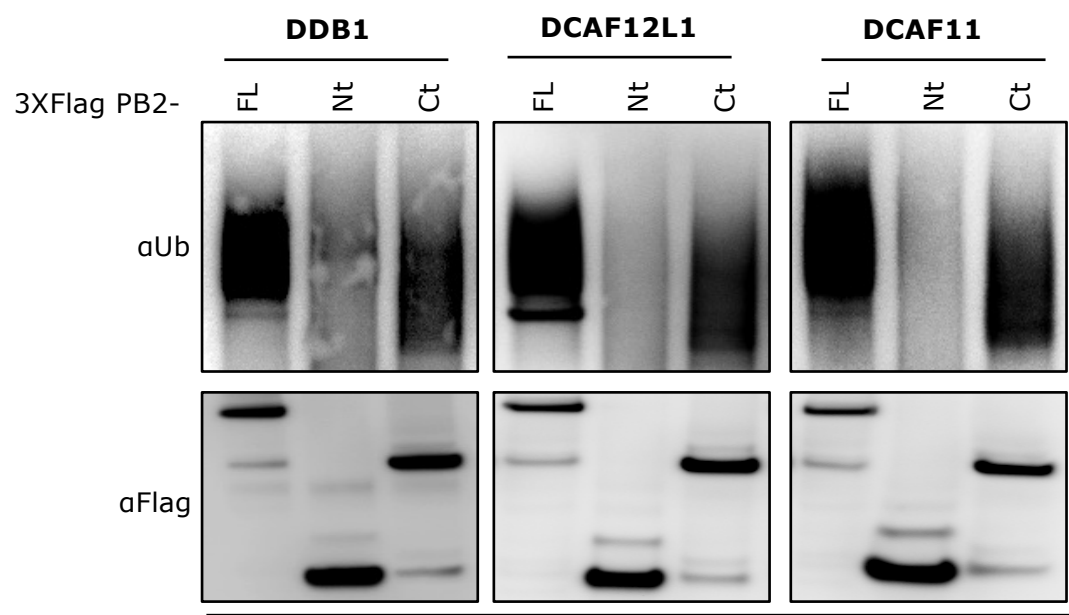

**IP Flag**

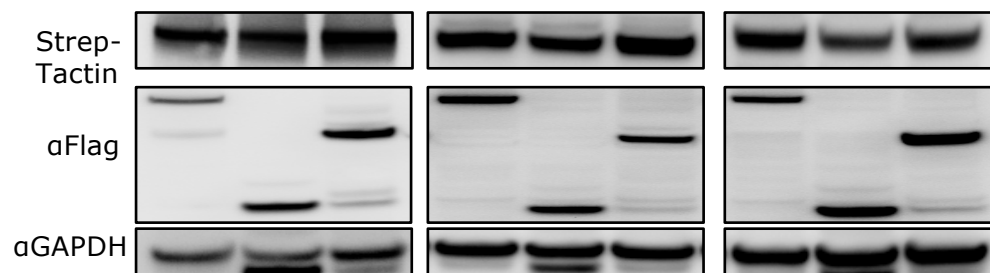

**Input**

Supplement: FIG S8 [file mBio.00305-20-sf008.pdf]

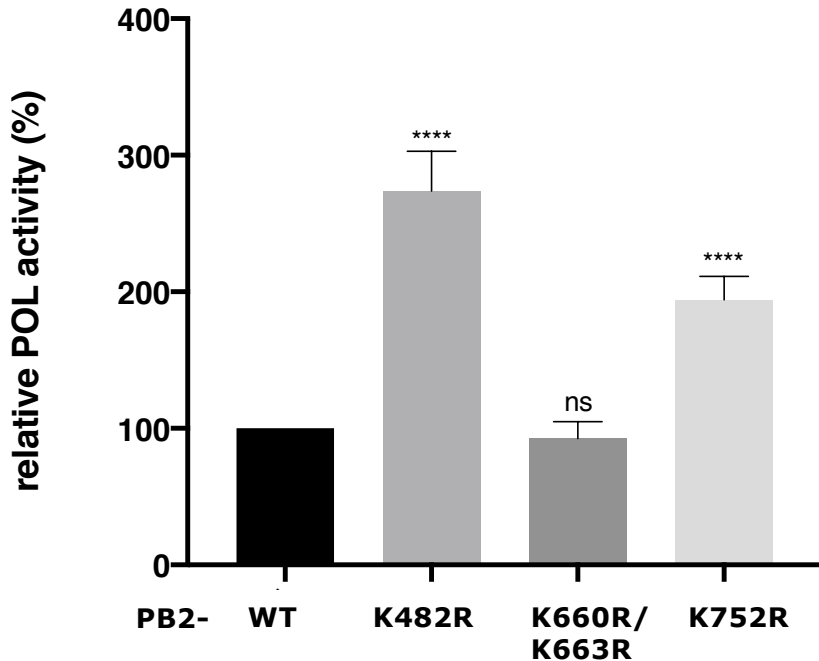

Supplement: FIG S9 [file mBio.00305-20-sf009.pdf]
