# Supplementary material for: Nonproteolytic K29-Linked Ubiquitination of the PB2 Replication Protein of Influenza A Viruses by Proviral Cullin 4-Based E3 Ligases
Source: mBio. 2020 Apr 7;11(2):e00305-20. doi: 10.1128/mBio.00305-20 (PMC7157767; doi:10.1128/mBio.00305-20)
Supplement: TABLE S1 [file mBio.00305-20-st001.pdf]

WSN

	24h		48h		72h	
	significance	Adjusted P Value	significance	Adjusted P Value	significance	Adjusted P Value
**DDB1**	**	0,0016	ns	0,0626	ns	0,5553
**DCAF12L1**	**	0,0031	*	0,0399	ns	0,7679
**DCAF11**	*	0,0176	**	0,0014	*	0,0117

H1N1pdm09

	24h		48h		72h	
	significance	Adjusted P Value	significance	Adjusted P Value	significance	Adjusted P Value
**DDB1**	***	0,0003	****	<0,0001	ns	0,1357
**DCAF12L1**	****	<0,0001	****	<0,0001	ns	0,0523
**DCAF11**	****	<0,0001	****	<0,0001	ns	0,1297

H3N2

	24h		48h		72h	
	significance	Adjusted P Value	significance	Adjusted P Value	significance	Adjusted P Value
**DDB1**	ns	0,1336	*	0,0165	ns	0,112
**DCAF12L1**	*	0,012	*	0,0253	ns	0,0792
**DCAF11**	*	0,0136	*	0,0111	ns	0,0503
